# Editorial: Cellular Therapies: Past, Present and Future

**DOI:** 10.3389/fimmu.2018.01966

**Published:** 2018-08-30

**Authors:** Ralf Dressel, Hildegard T. Greinix, Ernst Holler, Anne M. Dickinson

**Affiliations:** ^1^Institute of Cellular and Molecular Immunology, University Medical Centre Göttingen, Göttingen, Germany; ^2^Division of Haematology, Medical University of Graz, Graz, Austria; ^3^Department of Internal Medicine III, University Hospital of Regensburg, Regensburg, Germany; ^4^Hematological Sciences, Institute of Cellular Medicine, Newcastle University, Newcastle upon Tyne, United Kingdom

**Keywords:** animal models, biomarkers, genetic association, graft-vs.-host disease, graft-vs.-leukemia effects, hematopoietic stem cell transplantation, immunotherapies, reconstitution

This collection of review articles on hematopoietic stem cell transplantation (HSCT) has been compiled by enthusiastic post-graduate students within the Marie Curie Initial Training Network CELLEUROPE. It starts with two reviews of current research with a historical perspective and focus on either animal or human HSCT studies.

Boieri et al. summarize the importance of early animal experiments from the 1950's including graft-vs.-host disease (GvHD) a major complication of HSCT, initially termed “runt disease.” The article focuses on the contribution of animal experiments to the understanding of the pathophysiology of acute and chronic GvHD and the development of therapies to overcome these complications. It ends with a discussion of general advantages and limitations of animal models for studying GvHD.

The historic milestones of HSCT in man are reviewed by Juric et al. They describe the development of conditioning therapies enabling engraftment of HSC. The pivotal role of human leukocyte antigen (HLA) typing technologies for the introduction and continuous improvement of HSCT is explained. The shift from serological to molecular methods is discussed in view of their importance for the utilization of matched unrelated donors. Further parameters affecting HSCT outcome are introduced focusing on stem cell sources ranging from bone marrow to peripheral HSC and cord blood. The article closes with an outlook on new developments, such as the adoptive transfer of T cells engineered to express chimeric antigen receptors (CARs) directed against antigens present on leukemic cells.

Ghimire et al. discuss in detail the pathophysiology of GvHD explaining initiation and course of both the acute and chronic forms and introducing new strategies to limit these complications. Despite these efforts, especially chronic GvHD remains a major challenge that warrants further research.

The risk to elicit GvHD by transplantation of allogeneic T cells is balanced by their potential for profound graft-vs.-leukemia (GvL) effects, giving rise to curative therapies for malignancies. Dickinson et al. summarize clinical observations and experimental results demonstrating that allogeneic T cells in the graft reduce the risk of relapse of malignancy after HSCT. They describe the development of donor lymphocyte infusion (DLI) as a means to treat relapse. In addition, newer strategies are explained including the infusion of allogeneic mismatched natural killer (NK) cells or tumor antigen-specific T cells.

The reconstitution of the immune system after HSCT is reviewed by Ogonek et al.. It is of the utmost importance for the success of HSCT, that the various immune cell subsets recover and regain function in a timely manner. Neutrophils are the first cells that usually reappear within the first month after the conditioning therapy. They are followed in the first 3 months by NK cells and then by T cells. B cell recovery takes longer and may need up to 2 years post-HSCT. The reconstitution of regulatory T cells is explained focusing on factors which influence reconstitution, such as immunosuppressive treatment. Finally, the reconstitution of virus-specific T cells is reviewed in view of the clinical importance of these cells for avoiding complications such as CMV reactivation.

Biomarkers have gained much attention as they may predict the occurrence of GvHD and allow for risk-adapted treatment. Juric et al. review the recent developments and start with a discussion of cellular biomarkers, such as CD19^+^CD21^low^ B cells that are promising in predicting chronic GvHD. Besides cells, serum molecules have been reported to predict GvHD. In addition to proteins, miRNAs are potentially promising biomarkers for GvHD. Moreover, recent developments to identify biomarkers in urine by proteomic approaches are presented. Juric et al. close by discussing the challenge to validate and integrate the great variety of biomarkers that have been suggested during the last few years.

Gam et al. discuss the genetic associations of HSCT outcome focusing on non-histocompatibility genes and three specific examples. Firstly, it is discussed how polymorphisms of *FOXP3* and *FOXP3*-regulating microRNAs affect the risk of GvHD. The miR-155 and miR-146a regulatory network, their polymorphisms and role after HSCT is outlined as a second example. Polymorphisms of the *MICA* gene, which encodes a ligand of the activating NK cell receptor NKG2D, are introduced as a third example. Furthermore, mRNA and miRNA expression profiling studies aiming at the identification of HSCT associated risks are summarized. The review ends with a discussion of the few genome wide association studies, which have been performed so far to elucidate the role mainly of non-HLA polymorphisms in controlling the outcome of HSCT.

The final review by Reis et al. outlines recent developments in cellular immunotherapies for HSCT-associated complications. These include the transfer of mesenchymal stromal cells (MSCs) or MSC-derived extracellular vesicles to treat GvHD. Further complications are infections occurring in the immunocompromised patients. In recent years, strategies to employ anti-virus-specific T cells in the therapy of viral infections have been developed. Relapse is another outcome that can be targeted by cellular immunotherapies, e.g., with CAR T cells and in this chapter the challenges of these new and fascinating therapeutic strategies is discussed.

This collection of articles is dedicated to Professor Bent Rolstad (1947-2016), an enthusiastic and committed supervisor of two of the project's post-graduate students and Professor Jon van Rood (1926-2017), who was encouraging and supportive to the students in writing and revising the articles.

## Author contributions

RD drafted the manuscript, which AD, HG, and EH revised. All authors approved the final version.

### Conflict of interest statement

The authors declare that the research was conducted in the absence of any commercial or financial relationships that could be construed as a potential conflict of interest.

